# Mesial Stripping of Mandibular Deciduous Canines for Correction of Permanent Lateral Incisors

**DOI:** 10.5005/jp-journals-10005-144l

**Published:** 2017-02-27

**Authors:** Yahya B Nakhjavani, Farid B Nakhjavani, Ahmad Jafari

**Affiliations:** 1 Associate Professor, Department of Pediatric Dentistry, Tehran University of Medical Sciences, Tehran, Islamic Republic of Iran; 2 Lecturer, Department of Pediatric Dentistry, Tehran University of Medical Sciences, Tehran, Islamic Republic of Iran; 3 Associate Professor, Department of Community Oral Health, Tehran University of Medical Sciences, Tehran, Islamic Republic of Iran

**Keywords:** Crowding, Deciduous teeth, Mixed dentition, Proximal stripping.

## Abstract

**Aim:**

Stripping is a technique of creating space for correction of crowding by interproximal enamel reduction. This study sought to assess the efficacy of mesial stripping of mandibu-lar deciduous canines for correction of rotated and lingually erupted lateral incisors.

**Materials and methods:**

This clinical trial was performed on 42 patients with <3 mm mandibular anterior crowding. The required space was determined using the Moyers’ method and 3 mm of canine mesial surfaces was removed using a bur. Alginate impressions were made and the correction of crowding was evaluated until 5 months after the treatment. Data were analyzed using Fisher’s exact, Kruskal-Wallis, and Mann-Whitney U tests.

**Results:**

Mesial stripping of canines completely removed the crowding of anterior teeth; however; in a few cases, this correction was not complete, in which, the amount of space required was calculated to be near zero. Patient gender and occlusal relations had no significant effect on the correction of crowding; however, the amount of space required was significantly affected by the position of left lateral incisors (p < 0.001).

**Conclusion:**

Mesial stripping of deciduous canines is an effective technique to remove <3 mm crowding of buccally and lingually erupted permanent lateral incisors. Thus, stripping is recommended for space regaining and crowding correction.

**How to cite this article:**

Nakhjavani YB, Nakhjavani FB, Jafari A. Mesial Stripping of Mandibular Deciduous Canines for Correction of Permanent Lateral Incisors. Int J Clin Pediatr Dent 2017;10(3):229-233.

## INTRODUCTION

Space shortage for eruption of permanent teeth is a problem caused by tooth size/arch size discrepancy, premature loss of deciduous teeth, or primary tooth caries that leads to decreased arch length and insufficient space for eruption of permanent teeth.^1,2^ Space shortage usually manifests at an early age during the eruption of central and lateral incisors and the first signs of crowding often appear at this time;^3^ if not resolved, eruption of teeth in the following years will be impaired due to the lack of sufficient space.

Adequate space is required for leveling and aligning teeth in a crowded arch.^2^ After measuring the intercanine space and the sum of four anterior teeth, different treatment plans may be proposed to prevent crowding namely fixed or removable appliances, tooth extraction, distal movement of molar teeth, and reduction of mesiodistal widths of teeth.^1,2,4,5^

Air-rotor stripping (ARS) is one technique to create space by interproximal enamel reduction at areas with adequate enamel thickness during the mixed dentition period. This method was introduced by Sheridan as an alternative to tooth extraction for patients with mild to moderate crowding. He invented this technique by placing a 0.2 mm thick wire in the interdental space to prevent pulp injury and enamel reduction by a tungsten carbide bur.^6^ Mesial stripping of primary canines to eliminate space shortage by 3 to 8 mm and correction of contact areas have also been proposed.^3^ In this treatment, by mesial stripping of each canine tooth by 1.5 mm, 3 mm of space is gained. Moreover, ARS is also used for achieving other treatment goals, i.e., obtaining an ideal interincisal distance in dental discrepancies. Stripping of incisors can also correct the crowding.^7^ This technique is in fact a treatment modality for dental discrepancies without using orthodontic appliances and is usually indicated when tooth extraction is contraindicated.^7^ Due to its simplicity, it can be easily used in children aged 6 to 7 years who may have less cooperation in using orthodontic appliances.^3^ Due to its preventive nature, ARS can have beneficial effects on reducing the incidence of possible discrepancies. However, enamel stripping may increase the susceptibility of teeth to caries and in some cases rate of demineralization in the stripped enamel significantly increases compared with intact areas.^8^ Thus, this technique is suggested for use in patients with good oral hygiene and low risk of caries.

Nonextraction orthodontic treatments like ARS are becoming increasingly popular due to the existing con- troversies regarding the outcome of extraction orthodontic treatments, problems of tooth extraction in adult patients, and the unsuccessful results of overexpansion in nonextraction orthodontic patients.^9,10^ This study aimed to assess the efficacy of mesial stripping of mandibular primary canine teeth for correction of rotated or lingually erupted permanent lateral incisors.

**Fig. 1:**
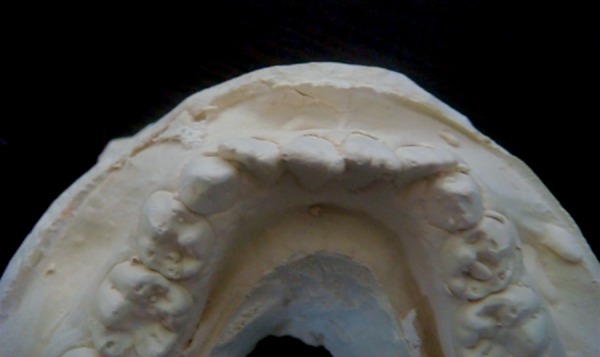
Position of lateral incisors which have erupted buccally

**Fig. 2:**
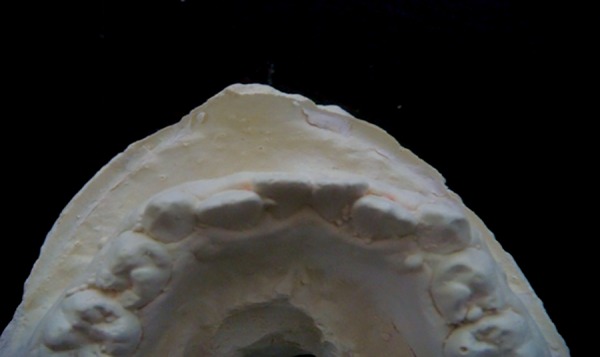
Position of lateral incisors which have erupted lingually

**Fig. 3:**
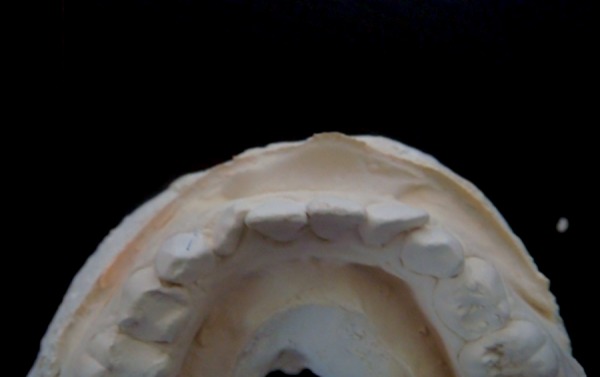
Position of lateral incisors which have erupted both buccally and lingually

## MATERIALS AND METHODS

This, before and after, clinical trial was performed on patients with crowding of permanent incisors and semi-erupted lateral incisors. Patients gave their written informed consent and attended all treatment sessions.

The amount of crowding was measured by a pedi-atric dentist using the Moyers’ method. The sum of the mesiodistal widths of four mandibular permanent incisors was measured and divided by 2. Using the obtained value and the Moyers’ table, the amount of required space was determined.^2^ Patients with more than 3 mm of space shortage or class III malocclusion were excluded from the study. Age, gender, amount of crowding, side of the jaw, and class of malocclusion were recorded in a special form. The position of lateral incisors is divided into three erupted categories as: (1) Buccally; (2) lingually; and (3) both buccally and lingually (Figs 1 to 3). According to the ARS technique, mesial surfaces of primary canines were stripped by 1.5 mm in such a way that the contact point was removed and the bur or a standard dental explorer could freely pass through the space between the lateral and canine teeth. All patients were followed up for 5 months and after completion of this time period, the amount of crowding was measured again as described above and recorded. The correlation of the before and after treatment amount of crowding with the type of occlusion, occlusal relation, and side of the jaw was statistically analyzed using paired t-test and the role of gender, class of malocclusion, occlusal relation, and side of the jaw in the crowding status was evaluated using Fisher’s exact test.

Figures 1 to 3 show the position of lateral incisors in the crowded arches.

## RESULTS

Initially, 52 patients were recruited in this study; out of which, 10 patients (19.2%) did not cooperate and were excluded. Thus, 42 patients were evaluated and followed up for 5 months. There were 18 boys (42.9%) and 24 girls (57.1%). Of the understudy subjects, 71.4% had normal occlusion, while 28.6% had class II malocclusion. In terms of the position of lateral incisors, they had erupted buc-cally in 33.3%, lingually in 40.5%, and both buccally and lingually in 26.2% of patients. In terms of space shortage, one-third of patients (n = 14) had less than 3 mm and 28 patients had 3 mm space shortage (Table 1).

**Table 1:** **Table 1:** Distribution of sample dental occlusion and measure of crowding based on gender

*Variables*		*Gender*		*Boys(%)*		*Girls (%)*	
Occlusion		Class I		15 (88.3%)		15 (62.5%)	
		Class II		3 (11.7%)		9 (37.5%)	
Crowding		<3 mm		6 (33.3%)		8 (33.3%)	
		3 mm		12 (66.7%)		16 (66.7%)	

**Table 2:** **Table 2:** Amount of crowding before and after treatment based on the position of lateral incisor and jaw quadrant

*Position of lateral incisor*		*Quadrant*		*Before treatment*		*After treatment*		*p-value*	
Buccal		Left		1/36 ± 0/16		0 ± 0		<0.001	
		Right		1/43 ± 0/19		0 ± 0		<0.001	
Lingual		Left		1/42 ± 0/14		0/06 ± 0/1		<0.001	
		Right		1/45 ± 0/15		0/05 ± 0/08		<0.001	
Buccal and lingual		Left		1/4 ± 0/1		0/08 ± 0/13		<0.001	
		Right		1/46 ± 0/15		0 ± 0		<0.001	

The amounts of crowding before and after stripping based on the position of lateral incisors and side of the jaw are shown in Table 2. As seen in Table 2, patients with left buccally erupted lateral incisors had 1.36 ± 0.16 mm space shortage, which decreased to zero by stripping (p < 0.001). Crowding was also completely resolved in patients with buccally erupted lateral incisor in the right quadrant. A significant reduction occurred in the amount of crowding in patients with lingually erupted lateral incisors in both right and left quadrants (p < 0.001). Crowding was completely removed in patients with buccally or lingually erupted left lateral incisors. In patients with right-side buccally or lingually erupted lateral incisors, the mean amount of crowding decreased from 1.4 mm before stripping to 0.08 mm after stripping (p < 0.001). Overall, the treatment response of buccally erupted teeth was more favorable than that of lingually erupted teeth.

Moreover, mesial stripping, regardless of the amount of space shortage, occlusal relations, quadrant of the jaw, dental space and gender, was effective for correction of crowding, while gender or occlusion had no significant effect on it (Table 3).

## DISCUSSION

Mesial stripping and interproximal enamel reduction of canine teeth had positive effects on correction of ≤3 mm crowding. In this study, mesial stripping completely removed the crowding in patients with buccally, lingually, or both buccally and lingually erupted lateral incisors; only in very few cases, the amount of crowding did not reach zero and a small crowding in the range of 0.06 to 0.1 mm remained.

**Table 3:** **Table 3:** Distribution of samples after correction of the crowding in gender, occlusion, and measure of crowding

*Variables*		*Crowding correction*		*No crowding*		*Crowding >0.5 mm*		*p-value*	
Gender		Boys		16		2		1	
		Girls		22		2			
Occlusion		Class I		26		4		0.308	
		Class II		12		0			
Crowding		<3 mm		14		0		0.283	
		3 mm		24		4			

In patients with class I and II malocclusion and buc-cally erupted left lateral incisors, the amount of space shortage was found to be zero in the final examination after treatment. It means that the crowding and space shortage were completely corrected. Moreover, the mean amount of space shortage in class II patients with lingually erupted lateral incisors was found to be zero at the fifth examination. This amount was 0.09 mm in class I patients with lingually erupted lateral incisors indicating incomplete correction of crowding. In class I patients with buccally-lingually erupted lateral incisors, the mean amount of space shortage at the fifth examination was 0.1 mm. This amount was zero in class II patients. These findings show that crowding in patients with class I and II malocclusion and all positions of lateral incisors was completely corrected by mesial stripping; but, in class I patients, complete correction only occurred for buccally erupted lateral incisors and in patients with lingually and lingually-buccally erupted lateral teeth; the amount of crowding significantly decreased, but it was not eliminated completely.

In the left quadrant of patients with <3 mm space shortage, the amount of crowding decreased to zero in all positions of lateral incisors (buccally, lingually, and buccally-lingually). However, in cases with 3 mm primary space shortage, crowding was corrected completely only in cases with buccally erupted lateral incisors. The mean amount of space shortage in the fifth examination of patients with 3 mm primary space shortage and lingually and lingually-buccally erupted lateral incisors was 0.06 and 0.1 mm respectively, which were close to zero.

In the right quadrant of patients with all positions of lateral incisors and different amounts of primary space shortage, crowding was completely corrected. However, in cases with 3 mm primary space shortage, crowding was only completely corrected in cases with buccally and buccally-lingually erupted laterals and the correction was not complete in cases with lingually erupted teeth. The mean amount of space shortage at the fifth examination in patients with incomplete resolution of crowding was found to be 0.05 mm, which was close to zero.

Evaluation of the correction of crowding in the right quadrant of patients with different positions of lateral incisors showed that in patients with buccally and buccally-lingually erupted teeth and class I and II malocclusion, crowding was corrected completely (the mean space shortage at the fifth examination = zero). However, in cases with class II malocclusion and lingually erupted lateral incisors, the crowding was not completely corrected (the mean space shortage at the fifth examination = 0.08 mm), although this amount was very small, approximating zero.

The results demonstrated that mesial stripping of mandibular primary canine teeth significantly improved crowding in patients with ≤3 mm primary space shortage indicating its efficacy.

Sheridan and Hastings^6^ in their study reported that enamel stripping established excellent occlusal and interincisal relations in class I malocclusion patients. The outcome of application of this technique in their study was beyond what was expected by the researchers. We only evaluated patients with ≤3 mm crowding, whereas Sheridan evaluated patients with greater amounts of crowding. Moreover, their applied technique was somehow different from ours. In another study, Germec; and Taner^11^ evaluated and compared the effects of extraction and nonextraction orthodontic treatments with ARS on patients with small crowding and reported that both techniques were suitable for correction of moderate crowding, and mesial stripping significantly decreased the treatment time. In their study, enamel stripping was 0.4 mm in the posterior segment and 0.25 mm in the anterior segment. In total, 1.5 mm of space was created, which was sufficient for the correction of 5.9 mm existing crowding (correction of crowding by 96%).

Before the introduction of full-arch bonding systems, the majority of mesial stripping techniques used to be performed in the incisors and before the bonding process. At present, due to the availability of bonding systems, interproximal stripping of teeth can be done at different times. The amount of interproximal stripping is directly correlated with the amount of crowding and space shortage in patients. For example, in cases with 3 mm of crowding, the amount of interproximal enamel reduction should be 3 mm. Space may be gained at any time and treatment may be done at any time in patients. Moreover, mesial stripping plays an undeniable role in improving the intercuspal space in patients. However, a hypothesis arises that stripping may cause tooth mass imbalance between the maxillary and mandibular arches. This issue was also discussed by Bolton.^12^ Nonetheless, mesial stripping should not be necessarily equal in the maxilla and mandible and these ratios may be changed in order to achieve acceptable intercuspal and intermaxillary relations. In the study by Sheridan and Hastings,^6^ the teeth were stripped in one jaw resulting in an optimal intercuspal relation. Considering the fact that in our study mesial stripping was done in the mesial surface of mandibular primary canines, the intercuspal relation was not evaluated after the treatment.

However, in order to improve the intercuspal relations in some cases, ARS should be necessarily performed in the opposing jaw as well due to differences in the mesiodistal widths of teeth.^13,14^ Lombardi^15^ showed that Bolton’s intermaxillary index did not have a significant association with the treatment outcome and it did not have sufficient accuracy for conversion of crowding to this index or redefining overbite and overjet. But, conflicting clinical evidences in this respect emphasize the need for further scrutiny.

Despite the positive results of mesial stripping for elimination of mild and moderate crowding, some clinicians still have doubts and concerns regarding the removal of relatively significant amounts of interproximal enamel especially in the posterior segments. However, it should be noted that damage to the tooth structure or periodontium following stripping has not been clinically confiremd.^6^ Furthermore, it seems that interproximal enamel stripping does not compromise the alveolar bone height or interproximal tissues, although mesial stripping changes the contact areas and decreases the interproximal tissue space. Some orthodontists may have some concerns regarding the long-term effects of this technique on the tooth supporting structures; however, this issue has not been confirmed either.^6^

Moreover, patients’ occlusion had no significant effect on the primary space shortage and correction of crowding in the right or left quadrants. Patients’ gender had no significant impact on the correction of crowding in the right or left quadrants either. Space shortage in the left side was significantly influenced by the position of lateral incisors (buccally, lingually, or buccally-lingually) but overjet and overbite had no significant effect on the amount of space shortage in the right or left quadrants. Thus, the obtained outcome in our study was only the result of mesial stripping of primary canine teeth, and except for the position of lateral incisor, no other variable played a role in this regard.

## CONCLUSION

Mesial stripping of mandibular primary canines resulted in complete correction of crowding in most cases; in the remaining ones, the amount of space shortage was decreased close to zero. Therefore, mesial stripping of primary canines is an effective treatment for patients with ≤3 mm crowding (except for the left quadrants where space shortage is significantly influenced by the position of lateral incisor). In conclusion, mesial stripping of primary canines is recommended for patients with ≤3 mm crowding.

## References

[1] Proffit WR., Fields HW Jr., Sarver DM. (2013). Contemporary orthodontics..

[2] Dean JA., Avery DR., McDonald RE. (2011). Dentistry for the child and adolescent..

[3] Al-Emran S (2007). Simple orthodontic tooth aligner. Saudi Dent J.

[4] Pinheiro ML (2002). Interproximal enamel reduction. World J Orthod.

[5] Moyers RE (1998). Handbook of orthodontics..

[6] Sheridan JJ, Hastings J (1992). Air-rotor stripping and lower incisor extraction treatment. J Clin Orthod.

[7] Rossouw PE, Tortorella A (2003). Enamel reduction procedures in orthodontic treatment. J Can Dent Assoc.

[8] Kilinc DD, Hamaci O (2009). Enamel surfaces with SEM after the application of different *in vivo* stripping methods. J Int Dent Med Res.

[9] Gianelly AA (2003). Arch width after extraction and nonextraction treatment. Am J Orthod Dentofacial Orthop.

[10] James RD (1998). A comparative study of facial profiles in extraction and nonextraction treatment. Am J Orthod Dentofacial Orthop.

[11] GermeÇ D, Taner TU (2008). Effects of extraction and nonextraction therapy with air-rotor stripping on facial esthetics in postado-lescent borderline patients. Am J Orthod Dentofacial Orthop.

[12] Munjal S, Duggal R, Kahlon SS, Bansal S (2010). Tooth size discrepancies in individuals presenting with different malocclusions. Indian J Dent Sci.

[13] Jadhav S, Vattipelli S, Pavitra M (2011). Interproximal enamel reduction in comprehensive orthodontic treatment: a review. Indian J Stomatol.

[14] Doris JM, Bernard BW, Kuftinec MM, Stom D (1981). A biometric study of tooth size and dental crowding. Am J Orthod.

[15] Lombardi AR (1972). Mandibular incisor crowding in completed cases. Am J Orthod.

